# Supplementary material for: Analysis of PPARγ Signaling Activity in Psoriasis
Source: Int J Mol Sci. 2021 Aug 10;22(16):8603. doi: 10.3390/ijms22168603 (PMC8395241; doi:10.3390/ijms22168603)
Supplement: Supplementary file 1 [file ijms-22-08603-s001.zip › Supplemental materials_Analysis of PPARg signaling activity in psoriasis/Pathway models/Models images and html files/Interleukin-17 and interleukin-22 signaling in psoriasis/6363.html]

IL10RB


# Protein IL10RB

|  |  |
| --- | --- |
| URN | urn:agi-llid:3588 |
| Total Entities | 0 |
| Connectivity | 293 |
| Name | IL10RB |
| Description | interleukin 10 receptor subunit beta |
| Notes | The protein encoded by this gene belongs to the cytokine receptor family. It is an accessory chain essential for the active interleukin 10 receptor complex. Coexpression of this and IL10RA proteins has been shown to be required for IL10-induced signal transduction. This gene and three other interferon receptor genes, IFAR2, IFNAR1, and IFNGR2, form a class II cytokine receptor gene cluster located in a small region on chromosome 21. [provided by RefSeq, Jul 2008] |
| Primary Cell Localization | Plasma membrane |
| Class | Receptor |
| ObjectType | Protein |

---

|  |  |
| --- | --- |
| Pathway | AHR in Intestinal Cell Antimicrobial Barrier Maintenance |
|  | anti-Inflammatory Function of Macrophage M2 Lineage |
|  | IL22 Induces Keratinocyte Proliferation in Psoriasis |
|  | Keratinocyte Activation in Psoriatic Arthritis |
|  | Gluten Impact on Neuronal System (Hypothesis) |
|  | IL10/STAT3 Signaling in M2 Macrophage and Retinal Angiogenesis |
|  | Alveolar Macrophage Negative Regulation Declined in COPD |
|  | STAT3 and NFkB Activate Inflammation-Induced Tumorigenesis |
|  | STAT3 Inhibits Maturation of Dendritic Cells and Facilitates Cancer Progression |
|  | STAT3 Facilitates the Function of Treg Cells and Cancer Progression |
|  | Myeloid Derived Suppressor Cells in Cancer Immune Escape |
|  | IFNLR1-> STAT/NFKB Signaling |
|  | IL22R -> STAT3 Signaling |
|  | IL10R -> STAT Signaling |
|  | IL10 Expression Targets |
|  | IL22 Expression Targets |
|  | IFN signaling in alveolar cells |
|  | 2\_Interleukin-17 and interleukin-22 signaling in psoriasis |
|  | 4\_1\_Polymorphisms associated with inflammatory bowel diseases |
|  | 1\_Inflammatory mediators increase the production of hepcidin (HAMP) |
|  | 2\_Defective tolerance of autoreactive T-cell in T1D |
|  | Interleukin-17 and interleukin-22 signaling in psoriasis |

---

|  |  |
| --- | --- |
| Group | Genes Highly-Expressed on Surface of Human Primary beta-Cell According to PMID:22460761 |

---

|  |  |
| --- | --- |
| MedScan ID | 3588 |

---

|  |  |
| --- | --- |
| LocusLink ID | 3588 |
|  | 16155 |
|  | 304091 |

---

|  |  |
| --- | --- |
| Alias | RGD1560373 |
|  | D21S58 |
|  | 6620401D04 |
|  | IL-10R subunit beta |
|  | antigen CDw210b |
|  | IL10r2 |
|  | CDw210b antigen |
|  | interleukin 10 receptor, beta |
|  | cytokine receptor family II, member IV |
|  | interleukin-10 receptor subunit 2 |
|  | cytokine receptor family 2 member 4 |
|  | CRF2-4 |
|  | interleukin IL10 receptor 2 |
|  | OTTHUMP00000221101 |
|  | interleukin IL-10 receptor beta |
|  | interleukin IL10R beta |
|  | interleukin IL10RB |
|  | IL10RB |
|  | interleukin IL-10R-B |
|  | PIR:G01418 |
|  | CRF2 IV |
|  | RIKEN cDNA 6620401D04 gene |
|  | IL-10 receptor subunit beta |
|  | CDW210B |
|  | IL-10 receptor beta |
|  | interleukin IL10r2 |
|  | D16H21S58 |
|  | cytokine receptor class-II CRF2 IV |
|  | D21S66 |
|  | CRFB4 |
|  | interleukin-10 receptor beta subunit |
|  | D21S58h |
|  | IL-10R-B |
|  | interleukin-10 receptor subunit beta |
|  | IL10R beta |
|  | AI528744 |
|  | interleukin 10 receptor 2 |
|  | IL-10R subunit 2 |
|  | 6620401D04Rik |
|  | cytokine receptor family II, member 4 |
|  | cytokine receptor class-II CRF2-4 |
|  | IL-10R2 |
|  | IL-10RB |
|  | cytokine receptor class-II member 4 |

---

|  |  |
| --- | --- |
| GO ID | 0004896 |
|  | 0004920 |
|  | 0038023 |
|  | 0019221 |
|  | 0051607 |
|  | 0006955 |
|  | 0006954 |
|  | 0046427 |
|  | 0007165 |
|  | 0016021 |
|  | 0032002 |
|  | 0005886 |
|  | 0016020 |
|  | 0007596 |
|  | 0070062 |
|  | 0004872 |

---

|  |  |
| --- | --- |
| KEGG ID | hsa:3588 |
|  | rno:304091 |
|  | mmu:16155 |

---

|  |  |
| --- | --- |
| Organism | Homo sapiens {Organism urn:agi-taxid:9606} |
|  | Mus musculus {Organism urn:agi-taxid:10090} |
|  | Rattus norvegicus {Organism urn:agi-taxid:10116} |
|  | Mus musculus |
|  | Homo sapiens |
|  | Rattus norvegicus |

---

|  |  |
| --- | --- |
| Mouse chromosome position | 16 52.87 cM |

---

|  |  |
| --- | --- |
| OMIM ID | 123889 |
|  | 610424 |
|  | 612567 |

---

|  |  |
| --- | --- |
| Rat chromosome position | 11q11 |

---

|  |  |
| --- | --- |
| Hugo ID | 5965 |
|  | HGNC:5965 |

---

|  |  |
| --- | --- |
| Human chromosome position | 21q22.11 |

---

|  |  |
| --- | --- |
| Swiss-Prot Accession | Q08334 |
|  | Q08334.2 |
|  | Q8VHM7 |
|  | Q3TU35 |
|  | Q61190.1 |
|  | D3ZM36 |
|  | Q9BUU4 |
|  | Q61190 |

---

|  |  |
| --- | --- |
| PIR ID | A47003 |

---

|  |  |
| --- | --- |
| GenBank ID | NC\_000021 |
|  | NM\_000628 |
|  | NP\_000619 |
|  | NG\_012089 |
|  | AP000295 |
|  | AP001716 |
|  | AY323826 |
|  | AAP72016 |
|  | CH471079 |
|  | EAX09840 |
|  | EAX09841 |
|  | U08988 |
|  | AAA86872 |
|  | AI025402 |
|  | AK124057 |
|  | BAC85761 |
|  | AK299961 |
|  | BAG61787 |
|  | AK313348 |
|  | AU133530 |
|  | BC001903 |
|  | AAH01903 |
|  | BT009777 |
|  | AAP88779 |
|  | JN591378 |
|  | AEX60807 |
|  | Z17227 |
|  | CAA78933 |
|  | Q08334 |
|  | NC\_000082 |
|  | NM\_008349 |
|  | NP\_032375 |
|  | XM\_006522924 |
|  | XP\_006522987 |
|  | AC152503 |
|  | AF440786 |
|  | AAL40943 |
|  | CH466602 |
|  | EDL03829 |
|  | EDL03830 |
|  | AF440787 |
|  | AAL40946 |
|  | AK018347 |
|  | AK040938 |
|  | AK134042 |
|  | BAE21991 |
|  | AK137922 |
|  | AK160991 |
|  | BAE36136 |
|  | AK177687 |
|  | AK179694 |
|  | AK181086 |
|  | AK183290 |
|  | AK195215 |
|  | AK196209 |
|  | AK201410 |
|  | AK208585 |
|  | AK213392 |
|  | BC145791 |
|  | AAI45792 |
|  | CK788837 |
|  | U53696 |
|  | AAC53062 |
|  | Q61190 |
|  | NC\_005110 |
|  | XM\_006248062 |
|  | XP\_006248124 |
|  | NM\_001107111 |
|  | NP\_001100581 |
|  | XM\_006248063 |
|  | XP\_006248125 |
|  | AC\_000079 |
|  | AABR07033630 |
|  | AABR07033631 |
|  | AABR07072220 |
|  | AAHX01068278 |
|  | AAHX01068279 |
|  | CH473989 |
|  | EDM10716 |
|  | EDM10717 |
|  | DQ892552 |
|  | AMYH02037735 |
|  | XP\_005261028 |
|  | XP\_005261027 |
|  | ABM86689 |
|  | AAHY01130186 |
|  | AAHY01130185 |
|  | AC\_000038 |
|  | DQ895763 |
|  | AABR06067655 |
|  | AABR06067654 |
|  | ABM83478 |
|  | AC\_000153 |
|  | L42243 |
|  | NC\_018932 |
|  | XM\_005260970 |
|  | XM\_005260971 |
|  | ABBA01057337 |
|  | ABBA01057336 |

---

|  |  |
| --- | --- |
| Swiss-Prot ID | I10R2\_HUMAN |

---

|  |  |
| --- | --- |
| Cell Localization | Membrane |
|  | Plasma membrane |

---

|  |  |
| --- | --- |
| Ensembl ID | ENSG00000243646 |
|  | ENSP00000290200.2 |
|  | ENST00000290200.7 |
|  | ENSMUSG00000022969 |
|  | ENSMUSP00000023691.5 |
|  | ENSMUST00000023691.11 |
|  | ENSRNOG00000028638 |
|  | ENSRNOP00000058062.3 |
|  | ENSRNOT00000061345.3 |
|  | ENSRNOT00000061345 |
|  | ENST00000290200 |
|  | ENST00000290200.6 |
|  | ENSP00000290200 |
|  | ENSRNOP00000058062 |
|  | ENSMUST00000023691 |
|  | ENSMUSP00000023691 |

---

|  |  |
| --- | --- |
| MGI ID | MGI:109380 |
|  | 109380 |

---

|  |  |
| --- | --- |
| RGD ID | 1560373 |

---

|  |  |
| --- | --- |
| Unigene ID | Hs.654593 |
|  | Mm.4154 |
|  | Rn.22261 |

---

|  |  |
| --- | --- |
| Homologene ID | 523 |

---
